# Supplementary material for: Egg Cooling After Oviposition Extends the Permissive Period for Microinjection-Mediated Genome Modification in Bombyx mori
Source: Int J Mol Sci. 2024 Nov 25;25(23):12642. doi: 10.3390/ijms252312642 (PMC11641327; doi:10.3390/ijms252312642)
Supplement: Supplementary file 1 [file ijms-25-12642-s001.zip › Supplementary caption_241124.pdf]

Table S1.

This experiment was performed to compare the efficiency of transgenic silkworm production between the conventional [63] and new manipulator. Microinjections were performed using eggs of 4 hAEL at 25 °C with the vector plasmid pBac(3xP3-DsRed2) and the helper plasmid pHAPIG in the final concentration of 0.2 μg/μL each. All G0 moths were crossed in a single mating manner with sibling mating. In each manipulator type, the experiments were performed three times. (a)–(d) represent column symbol. Conventional, oil hydraulic manipulator; New, semi-automatic electric manipulator; The conventional manipulator details are described in [63].

Table S2.

In total, 384 eggs were injected in each experiment, corresponding to the experiments (#1 to #3) in Table 1. The total time for injection was delayed in the experiment #2 in the new manipulator due to unfortunate capillary clogging.

Figure S1

The *BmBLOS2* knock-out phenotype at the 4th instar larvae. a, not injected; b–h, injected using the eggs stored for 24 h at the different temperatures (b, -20 °C; c, -3 °C; d, 0 °C; e, 5 °C; f, 10 °C; g, 15 °C; h, 20 °C); i–l, injected using the eggs stored at 10 °C for the different times (i, 48 h; j, 72 h; k, 96 h; l, 120 h); N/A, not applicable.

Figure S2. The microinjection system using a semi-automatic electric manipulator. (A) Photograph and illustration of the whole microinjection system. The red part shows a newly introduced manipulator device, M300, and the blue one is an adjustor of the needle and glass capillary shown in "D." (B) The magnified figure of the red part in "A." The needle and capillary can be three-dimensionally moved. (C) An image of the operation with the controller, operation board, of manipulator device. The computer can memorize each position of both the needle and the capillary and switch them to each position with the red buttons. (D) Photograph and illustration of the needle and capillary adjustor, HD-21. This is used during the replacement of capillaries. Screw a, b, and c can adjust the position of the glass capillary, for the x-, y-, and z-axis, and screw d can adjust the position of the needle for the z-axis. needle; tungsten needle, capillary; glass capillary.

Figure S3: Appearance of microinjection into an egg. Tn, tungsten needle; Gc, glass capillary.
