# Supplementary material for: Egg Cooling After Oviposition Extends the Permissive Period for Microinjection-Mediated Genome Modification in Bombyx mori
Source: Int J Mol Sci. 2024 Nov 25;25(23):12642. doi: 10.3390/ijms252312642 (PMC11641327; doi:10.3390/ijms252312642)
Supplement: Supplementary file 1 [file ijms-25-12642-s001.zip › Supplementary tables_241124.pdf]

Table S1. The efficiency of transgenic silkworm production between the conventional and new manipulators.

Manipulator type	Experimental No.	(a) No. of injected eggs	(b) No. of hatched eggs (b/a*100)	Avg. pct. of the column (b) (Mean ± SE)	(c) No. of G_1 broods	(d) No. of G_1 DsRed2-positive broods (d/c*100)	Avg. pct. Of the column (d) (Mean ± SE)
Conventional	#1	384	210 (54.7%)	61.6% ± 4.1%	61	4 (6.6%)	20.4% ± 6.9%
	#2	384	265 (69.0%)		81	23 (28.4%)	
	#3	384	235 (61.2%)		80	21 (26.3%)	
New	#1	384	227 (59.1%)	64.1% ± 4.0%	72	23 (31.9%)	27.7% ± 4.3%
	#2	384	235 (61.2%)		78	25 (32.1%)	
	#3	384	277 (72.1%)		99	19 (19.2%)	

Table S2. Time comparison with the microinjection between the conventional and new manipulators.

Manipulator types	Experimental no.	Total time for injection
Conventional	#1	94 min.
	#2	94 min.
	#3	95 min.
New	#1	59 min.
	#2	81 min.
	#3	54 min.
