# Supplementary figures and images for: Egg Cooling After Oviposition Extends the Permissive Period for Microinjection-Mediated Genome Modification in Bombyx mori
Source: Int J Mol Sci. 2024 Nov 25;25(23):12642. doi: 10.3390/ijms252312642 (PMC11641327; doi:10.3390/ijms252312642)

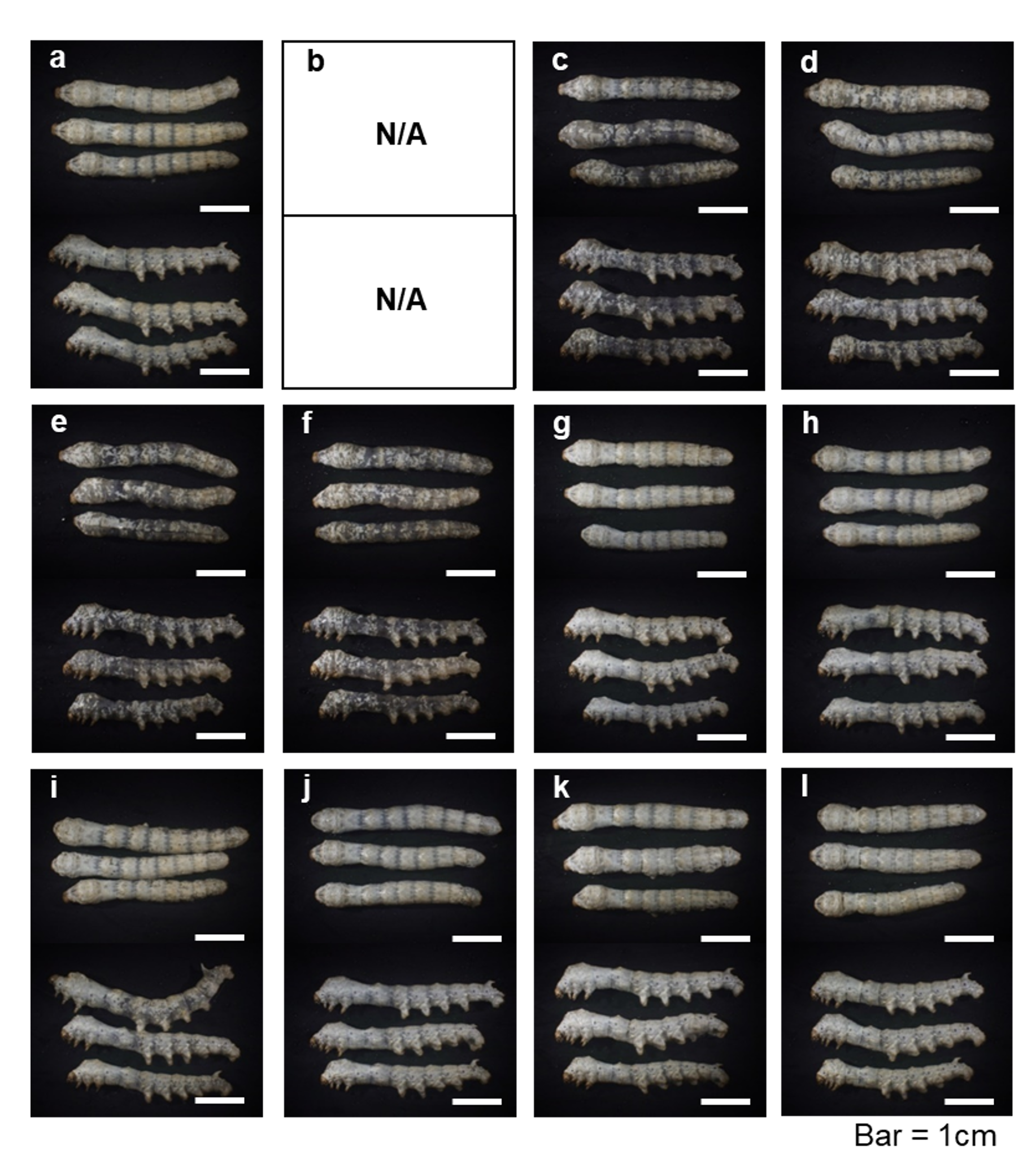

Supplement: Supplementary file 1 [file ijms-25-12642-s001.zip › Figure S1_241109.png]

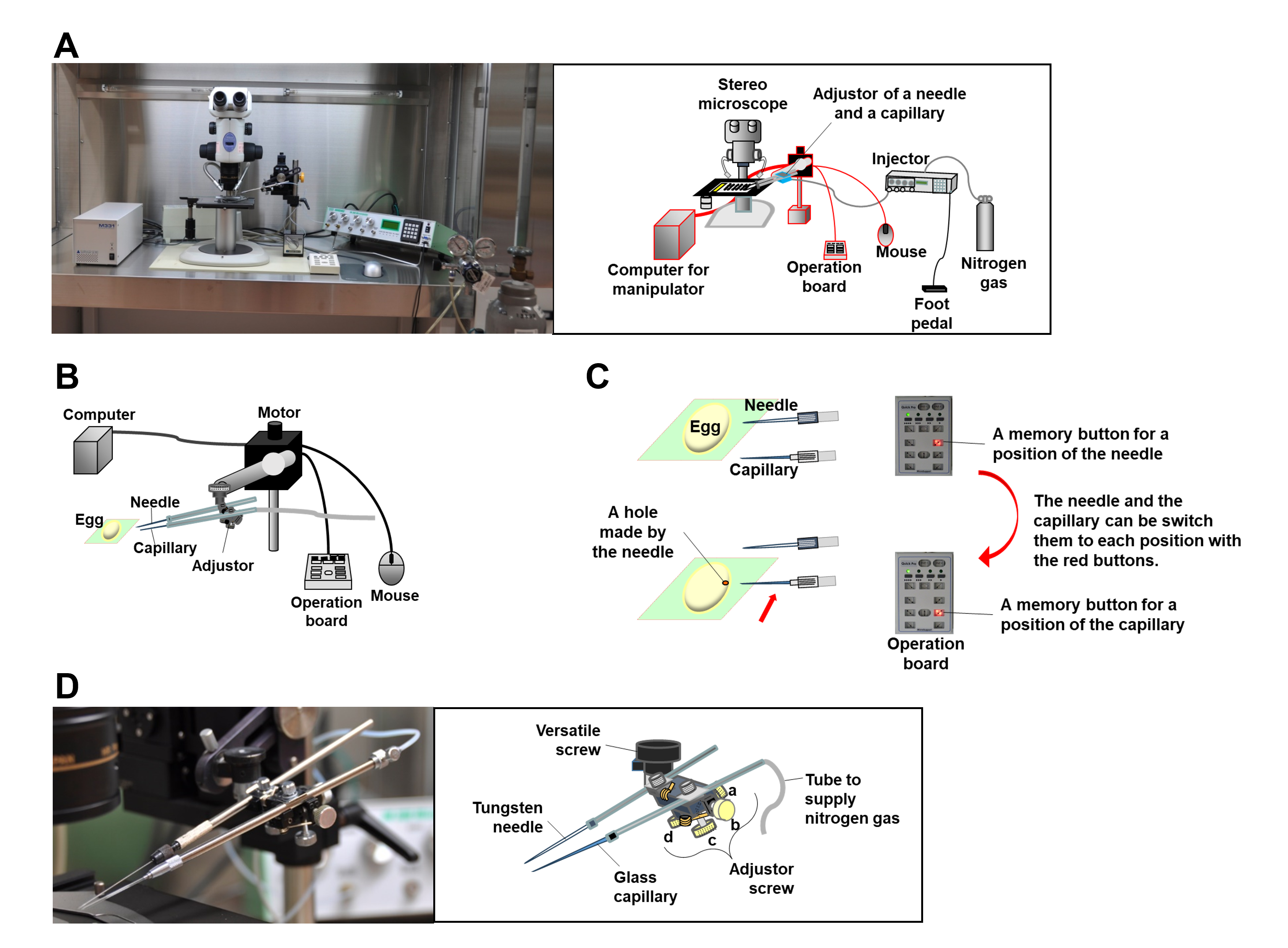

Supplement: Supplementary file 1 [file ijms-25-12642-s001.zip › Figure S2_241109.png]

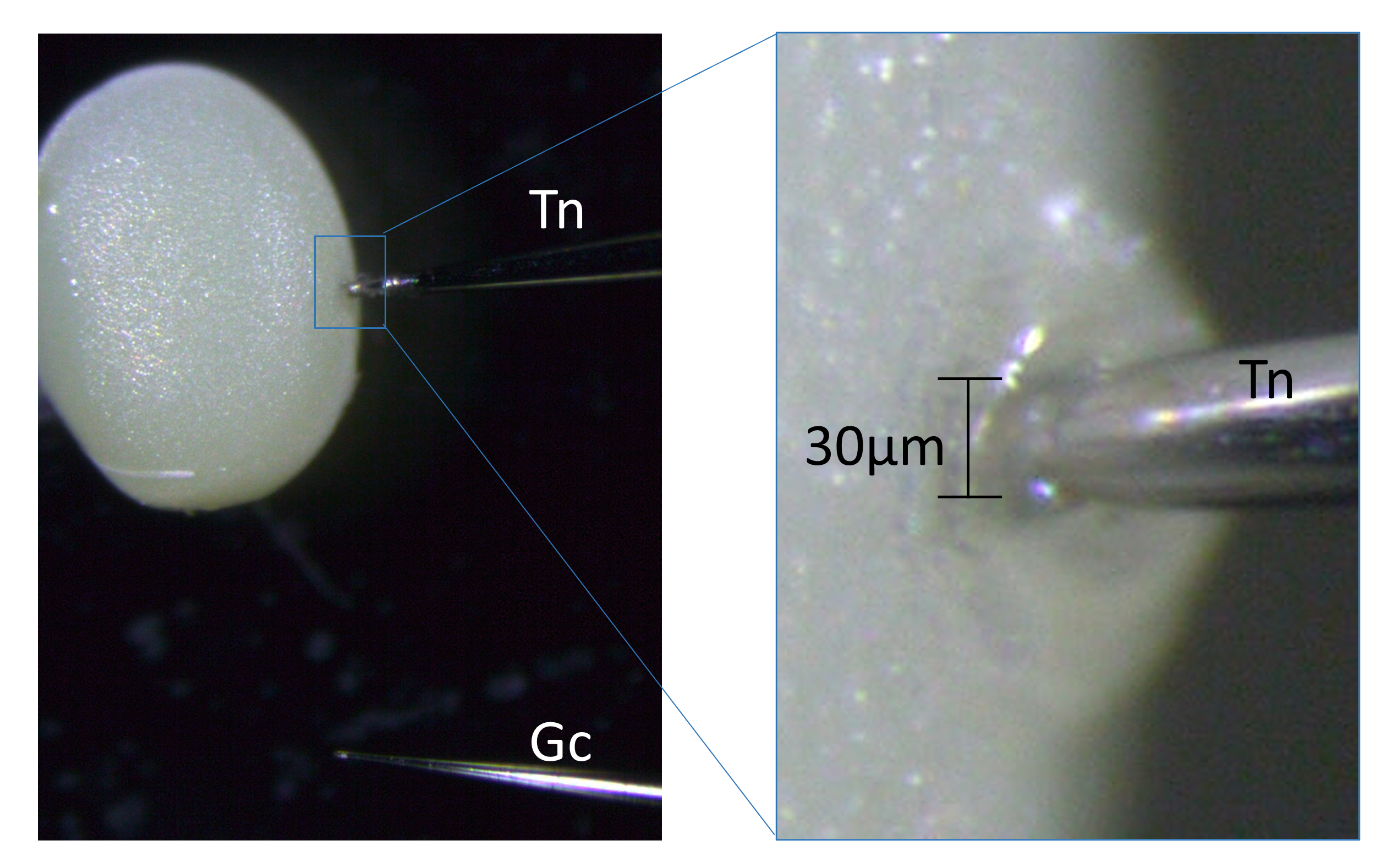

Supplement: Supplementary file 1 [file ijms-25-12642-s001.zip › Figure S3_241109.png]
